# Development of long-acting ARV’s

**DOI:** 10.1186/1471-2334-14-S2-S11

**Published:** 2014-05-23

**Authors:** Marta Boffito

**Affiliations:** 1 Chelsea and Westminster Hospital, Saint Stephen’s Center, London, UK

## 

Research on improved treatment of HIV infection and pre-exposure prophylaxis (PrEP) continues. Poor adherence to treatment is the critical risk factor for virologic failure and resistance development, and long-acting formulations of anti-HIV medications that need only infrequent dosing may facilitate long-term therapeutic responses. However, such formulations and long-term persistence of drug are associated with challenges, such as safety and selective pressure of the drug in case of resistance.

Furthermore, to prevent transmission of HIV, long-acting injectable formulations of ARV agents with infrequent dosing may be advantageous over daily oral drug intake. However, the knowledge on protective drug concentrations and frequency of dosing is poor to date and implementation globally is challenging.

